# 2-[6,8-Dibromo-3-(4-hy­droxy­cyclo­hex­yl)-1,2,3,4-tetra­hydro­quinazolin-2-yl]phenol methanol 0.25-solvate

**DOI:** 10.1107/S1600536811007987

**Published:** 2011-03-09

**Authors:** Zhi-Gang Wang, Rong Wang, Feng Zhi, Ming-Li Wang

**Affiliations:** a Department of Respiratory Medicine, Third Affiliated Hospital of Soochow University, Changzhou 213003, People’s Republic of China; b Modern Medical Research Center, Third Affiliated Hospital of Soochow University, Changzhou 213003, People’s Republic of China; c Department of Pharmacy, Third Affiliated Hospital of Soochow University, Changzhou 213003, People’s Republic of China

## Abstract

The title compound, C_20_H_22_Br_2_N_2_O_2_·0.25CH_4_O, was synthesized by the condensation reaction of salicyl­aldehyde with 4-(2-amino-3,5-dibromo­benzyl­amino)­cyclo­hexa­nol in methanol. There are four independent main mol­ecules and two half-occupied methanol solvent mol­ecules in the asymmetric unit. The dihedral angles between the two benzene rings in the four mol­ecules are 87.8 (6), 86.6 (6), 89.3 (6) and 83.1 (6)°. Each mol­ecule features an intra­molecular O—H⋯N hydrogen bond and a short N—H⋯Br link. In the crystal components are linked by O—H⋯O hydrogen bonds.

## Related literature

For details of the pharmaceutical uses of ambroxol, systematic name 4-(2-amino-3,5-dibromo­benzyl­amino)­cyclo­hexa­nol, a compound closely related to the title compound, see: Gaida *et al.* (2005 ▶); Lee *et al.* (2004 ▶). For the structures of similar compounds, see: Wang *et al.* (2009 ▶, 2010 ▶). For standard bond-length data, see: Allen *et al.* (1987 ▶).
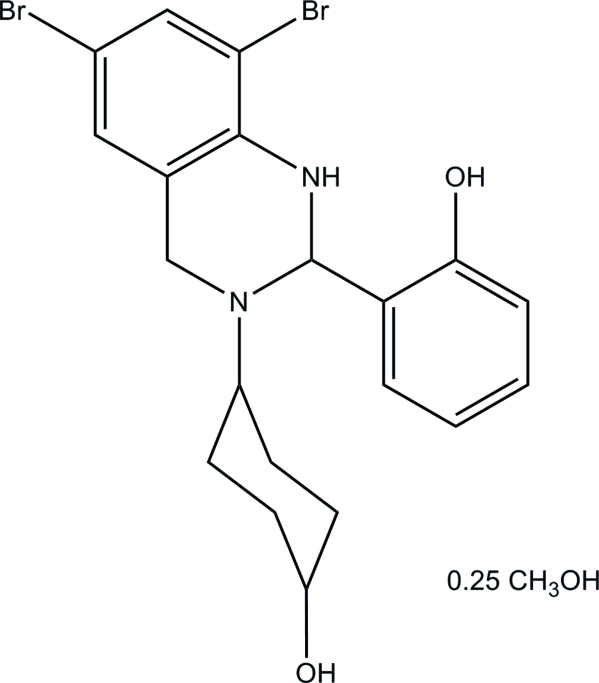


## Experimental

### 

#### Crystal data

C_20_H_22_Br_2_N_2_O_2_·0.25CH_4_O
*M* *_r_* = 490.23
Triclinic, 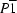

*a* = 11.733 (2) Å
*b* = 16.831 (3) Å
*c* = 21.721 (4) Å
α = 94.69 (3)°
β = 96.88 (3)°
γ = 99.44 (3)°
*V* = 4178.4 (14) Å^3^
*Z* = 8
Mo *K*α radiation
μ = 3.90 mm^−1^
*T* = 298 K
0.23 × 0.23 × 0.20 mm

#### Data collection

Bruker SMART CCD diffractometer
Absorption correction: multi-scan (*SADABS*; Sheldrick, 1996 ▶) *T*_min_ = 0.468, *T*_max_ = 0.509
46008 measured reflections
17573 independent reflections
6508 reflections with *I* > 2σ(*I*)
*R*_int_ = 0.108

#### Refinement

*R*[*F* ^2^ > 2σ(*F* ^2^)] = 0.087
*wR*(*F* ^2^) = 0.199
*S* = 0.95
17573 reflections
1001 parameters
38 restraints
H atoms treated by a mixture of independent and constrained refinement
Δρ_max_ = 1.06 e Å^−3^
Δρ_min_ = −0.78 e Å^−3^

### 

Data collection: *SMART* (Bruker, 2002 ▶); cell refinement: *SAINT* (Bruker, 2002 ▶); data reduction: *SAINT*; program(s) used to solve structure: *SHELXS97* (Sheldrick, 2008 ▶); program(s) used to refine structure: *SHELXL97* (Sheldrick, 2008 ▶); molecular graphics: *SHELXTL* (Sheldrick, 2008 ▶); software used to prepare material for publication: *SHELXTL*.

## Supplementary Material

Crystal structure: contains datablocks global, I. DOI: 10.1107/S1600536811007987/hb5804sup1.cif

Structure factors: contains datablocks I. DOI: 10.1107/S1600536811007987/hb5804Isup2.hkl

Additional supplementary materials:  crystallographic information; 3D view; checkCIF report

## Figures and Tables

**Table 1:** Hydrogen-bond geometry (Å, °)

*D*—H⋯*A*	*D*—H	H⋯*A*	*D*⋯*A*	*D*—H⋯*A*
O1—H1⋯N4	0.82	1.93	2.643 (8)	145
N1—H1*A*⋯Br2	0.91 (8)	2.69 (7)	3.081 (7)	107 (6)
O2—H2⋯O9^i^	0.82	1.85	2.660 (15)	167
O3—H3⋯N2	0.82	1.93	2.661 (9)	148
N3—H3*B*⋯Br4	0.91 (8)	2.77 (8)	3.097 (7)	103 (5)
O4—H4⋯O6^ii^	0.82	2.13	2.721 (10)	129
O5—H5⋯N6	0.82	1.95	2.650 (9)	143
N5—H5*B*⋯Br5	0.90 (6)	2.77 (8)	3.110 (7)	104 (6)
O6—H6⋯O8^iii^	0.82	1.95	2.735 (9)	160
O7—H7⋯N8	0.85 (6)	1.99 (8)	2.717 (9)	142 (6)
N7—H7*B*⋯Br7	0.90 (6)	2.61 (8)	3.100 (7)	115 (6)
O8—H8⋯O2^iv^	0.84 (9)	1.90 (8)	2.713 (11)	162 (8)
O9—H9⋯O10^v^	0.82	2.18	2.94 (2)	156

Symmetry codes: (i) 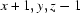; (ii) 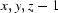; (iii) 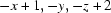; (iv) 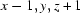; (v) 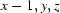.

**Fig. 1.**
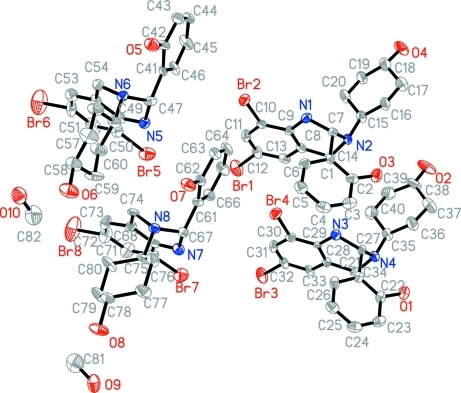
The asymmetric unit of the title compound showing 30% probability ellipsoids. H atoms bonded to C atoms are not shown.

## supplementary crystallographic information

### Comment

Ambroxol [or 4-(2-amino-3,5-dibromobenzylamino)cyclohexanol] is an expectorant agent which leads to bronchial secretion due to its  mucolytic properties (Gaida *et al.*, 2005; Lee *et al.*, 2004). Recently, we reported the crystal structures of two derivatives of Ambroxol (Wang *et al.*, 2009; Wang *et al.*, 2010). In this paper, the crystal structure of the title compound, derived from the condensation reaction of salicylaldehyde with 4-(2-amino-3,5-dibromobenzylamino)cyclohexanol in methanol, is reported.

There are four independent molecules and two methanol molecules in the asymmetric unit of the title compound, Fig. 1. The dihedral angles between the two benzene rings in the four molecules are 87.8 (6), 86.6 (6), 90.7 (6), and 96.9 (6)°, respectively. The cyclohexyl rings adopt chair configurations. All  bond lengths are within normal ranges (Allen *et al.*, 1987).

### Experimental

Salicylaldehyde (1.0 mol, 122.1 mg) and 4-(2-amino-3,5-dibromobenzylamino)cyclohexanol (1.0 mmol, 378.1 mg) were dissolved in methanol (30 ml). The mixture was stirred at room temperature to give a clear colorless solution. Colourless blocks of the title compound were formed by gradual evaporation of the solvent for a few days at room temperature.

### Refinement

H1A, H3B, H5B, H7B, and H7 atoms were located from a difference Fourier map and refined isotropically, with N—H and O—H distances restrained to 0.90 (1) and 0.85 (1) Å, respectively. The other H atoms were included in calulated positions with, with C—H = 0.93–0.98 Å, and O—H = 0.82 Å, and with *U*_iso_(H) = 1.2*U*_eq_(C) or 1.5U_eq_(O and methyl C).

### Crystal data

**Table:**

C_20_H_22_Br_2_N_2_O_2_·0.25CH_4_O	*Z* = 8
*M**_r_* = 490.23	*F*(000) = 1972
Triclinic, *P*1	*D*_x_ = 1.559 Mg m^−^^3^
Hall symbol: -P 1	Mo *K*α radiation, λ = 0.71073 Å
*a* = 11.733 (2) Å	Cell parameters from 2369 reflections
*b* = 16.831 (3) Å	θ = 2.5–24.3°
*c* = 21.721 (4) Å	µ = 3.90 mm^−^^1^
α = 94.69 (3)°	*T* = 298 K
β = 96.88 (3)°	Block, colorless
γ = 99.44 (3)°	0.23 × 0.23 × 0.20 mm
*V* = 4178.4 (14) Å^3^	

### Data collection

**Table:**

Bruker SMART CCD diffractometer	17573 independent reflections
Radiation source: fine-focus sealed tube	6508 reflections with *I* > 2σ(*I*)
graphite	*R*_int_ = 0.108
ω scans	θ_max_ = 27.0°, θ_min_ = 2.3°
Absorption correction: multi-scan (*SADABS*; Sheldrick, 1996)	*h* = −14→14
*T*_min_ = 0.468, *T*_max_ = 0.509	*k* = −21→21
46008 measured reflections	*l* = −27→27

### Refinement

**Table:**

Refinement on *F*^2^	Primary atom site location: structure-invariant direct methods
Least-squares matrix: full	Secondary atom site location: difference Fourier map
*R*[*F*^2^ > 2σ(*F*^2^)] = 0.087	Hydrogen site location: inferred from neighbouring sites
*wR*(*F*^2^) = 0.199	H atoms treated by a mixture of independent and constrained refinement
*S* = 0.95	*w* = 1/[σ^2^(*F*_o_^2^) + (0.0601*P*)^2^] where *P* = (*F*_o_^2^ + 2*F*_c_^2^)/3
17573 reflections	(Δ/σ)_max_ = 0.001
1001 parameters	Δρ_max_ = 1.06 e Å^−^^3^
38 restraints	Δρ_min_ = −0.78 e Å^−^^3^

### Special details

**Table:**

Geometry. All esds (except the esd in the dihedral angle between two l.s. planes) are estimated using the full covariance matrix. The cell esds are taken into account individually in the estimation of esds in distances, angles and torsion angles; correlations between esds in cell parameters are only used when they are defined by crystal symmetry. An approximate (isotropic) treatment of cell esds is used for estimating esds involving l.s. planes.
Refinement. Refinement of F^2^ against ALL reflections. The weighted R-factor wR and goodness of fit S are based on F^2^, conventional R-factors R are based on F, with F set to zero for negative F^2^. The threshold expression of F^2^ > 2sigma(F^2^) is used only for calculating R-factors(gt) etc. and is not relevant to the choice of reflections for refinement. R-factors based on F^2^ are statistically about twice as large as those based on F, and R- factors based on ALL data will be even larger.

### Fractional atomic coordinates and isotropic or equivalent isotropic displacement parameters (Å^2^)

**Table:**

	*x*	*y*	*z*	*U*_iso_*/*U*_eq_	Occ. (<1)
Br1	0.50780 (11)	0.54851 (6)	0.41605 (5)	0.0858 (4)	
Br2	0.75088 (8)	0.28881 (6)	0.44920 (4)	0.0564 (3)	
Br3	1.02222 (11)	0.55820 (6)	0.42630 (5)	0.0768 (4)	
Br4	1.26724 (8)	0.29701 (6)	0.44757 (4)	0.0573 (3)	
Br5	0.73267 (9)	0.40022 (6)	0.68344 (5)	0.0733 (3)	
Br6	1.12176 (15)	0.56107 (7)	0.85040 (8)	0.1393 (7)	
Br7	0.22232 (9)	0.41215 (6)	0.68357 (5)	0.0680 (3)	
Br8	0.59680 (15)	0.58398 (7)	0.85557 (8)	0.1367 (7)	
O1	0.7323 (5)	0.0798 (3)	0.2677 (3)	0.0541 (16)	
H1	0.7805	0.1076	0.2504	0.081*	
O2	1.1310 (8)	0.2056 (5)	0.0423 (4)	0.109 (3)	
H2	1.0954	0.2393	0.0275	0.164*	
O3	0.2270 (5)	0.0715 (4)	0.2618 (3)	0.0570 (17)	
H3	0.2705	0.1041	0.2455	0.085*	
O4	0.6491 (7)	0.1764 (5)	0.0424 (3)	0.085 (2)	
H4	0.7056	0.1542	0.0391	0.128*	
O5	1.0710 (5)	0.0754 (4)	0.7097 (3)	0.0603 (17)	
H5	1.0295	0.0842	0.7364	0.090*	
O6	0.7231 (6)	0.0560 (4)	0.9749 (3)	0.075 (2)	
H6	0.7517	0.0184	0.9883	0.113*	
O7	0.5483 (6)	0.0782 (4)	0.7181 (3)	0.0605 (17)	
O8	0.2347 (6)	0.0897 (4)	0.9893 (3)	0.073 (2)	
N1	0.5676 (6)	0.2056 (4)	0.3396 (3)	0.0406 (17)	
N2	0.4082 (5)	0.1867 (4)	0.2547 (3)	0.0368 (16)	
N3	1.0712 (6)	0.2146 (4)	0.3426 (3)	0.0405 (17)	
N4	0.9067 (5)	0.1991 (4)	0.2622 (3)	0.0369 (16)	
N5	0.8385 (6)	0.2493 (4)	0.7178 (3)	0.0430 (17)	
N6	0.9564 (5)	0.1679 (4)	0.7749 (3)	0.0341 (16)	
N7	0.3360 (6)	0.2657 (4)	0.7195 (3)	0.0466 (18)	
N8	0.4575 (5)	0.1926 (4)	0.7824 (3)	0.0393 (17)	
C1	0.3768 (7)	0.1178 (4)	0.3488 (4)	0.0338 (19)	
C2	0.2633 (8)	0.0780 (5)	0.3238 (5)	0.044 (2)	
C3	0.1865 (8)	0.0458 (4)	0.3639 (5)	0.052 (3)	
H3A	0.1120	0.0187	0.3480	0.063*	
C4	0.2228 (9)	0.0548 (5)	0.4272 (5)	0.054 (3)	
H4A	0.1713	0.0338	0.4536	0.065*	
C5	0.3321 (9)	0.0935 (5)	0.4524 (4)	0.055 (3)	
H5A	0.3552	0.0990	0.4952	0.066*	
C6	0.4061 (7)	0.1239 (5)	0.4130 (4)	0.047 (2)	
H6A	0.4805	0.1503	0.4301	0.057*	
C7	0.4654 (7)	0.1496 (5)	0.3066 (4)	0.037 (2)	
H7A	0.4938	0.1027	0.2881	0.044*	
C8	0.4625 (7)	0.3155 (4)	0.3258 (3)	0.036 (2)	
C9	0.5528 (6)	0.2828 (4)	0.3580 (4)	0.0330 (19)	
C10	0.6297 (7)	0.3326 (5)	0.4049 (4)	0.039 (2)	
C11	0.6164 (8)	0.4108 (5)	0.4212 (4)	0.052 (2)	
H11	0.6679	0.4428	0.4530	0.062*	
C12	0.5289 (8)	0.4412 (5)	0.3912 (4)	0.049 (2)	
C13	0.4520 (7)	0.3943 (5)	0.3435 (4)	0.047 (2)	
H13	0.3922	0.4163	0.3230	0.057*	
C14	0.3733 (7)	0.2620 (5)	0.2785 (4)	0.047 (2)	
H14A	0.3024	0.2481	0.2971	0.057*	
H14B	0.3553	0.2923	0.2437	0.057*	
C15	0.4654 (8)	0.1917 (5)	0.1979 (4)	0.047 (2)	
H15	0.4097	0.2119	0.1684	0.056*	
C16	0.4707 (8)	0.1082 (5)	0.1677 (4)	0.056 (3)	
H16A	0.3942	0.0745	0.1631	0.067*	
H16B	0.5243	0.0832	0.1940	0.067*	
C17	0.5117 (9)	0.1145 (6)	0.1033 (4)	0.070 (3)	
H17A	0.5234	0.0615	0.0870	0.084*	
H17B	0.4505	0.1301	0.0752	0.084*	
C18	0.6187 (9)	0.1720 (6)	0.1038 (5)	0.070 (3)	
H18	0.6809	0.1512	0.1283	0.084*	
C19	0.6130 (8)	0.2539 (5)	0.1356 (4)	0.062 (3)	
H19A	0.5593	0.2794	0.1097	0.074*	
H19B	0.6894	0.2877	0.1401	0.074*	
C20	0.5737 (9)	0.2483 (5)	0.1989 (4)	0.064 (3)	
H20A	0.6343	0.2317	0.2268	0.077*	
H20B	0.5634	0.3016	0.2155	0.077*	
C21	0.8835 (7)	0.1260 (4)	0.3539 (4)	0.037 (2)	
C22	0.7711 (8)	0.0852 (5)	0.3305 (4)	0.044 (2)	
C23	0.6981 (8)	0.0501 (5)	0.3688 (5)	0.052 (2)	
H23	0.6245	0.0221	0.3520	0.063*	
C24	0.7325 (9)	0.0556 (5)	0.4320 (5)	0.057 (3)	
H24	0.6813	0.0328	0.4579	0.068*	
C25	0.8405 (9)	0.0942 (5)	0.4566 (4)	0.055 (3)	
H25	0.8641	0.0971	0.4993	0.066*	
C26	0.9148 (8)	0.1290 (5)	0.4190 (4)	0.048 (2)	
H26	0.9887	0.1556	0.4367	0.057*	
C27	0.9673 (7)	0.1606 (4)	0.3101 (4)	0.036 (2)	
H27	0.9940	0.1147	0.2891	0.043*	
C28	0.9668 (7)	0.3264 (4)	0.3369 (4)	0.038 (2)	
C29	1.0599 (6)	0.2932 (4)	0.3632 (4)	0.0319 (19)	
C30	1.1402 (7)	0.3414 (5)	0.4079 (4)	0.041 (2)	
C31	1.1315 (7)	0.4202 (5)	0.4270 (4)	0.046 (2)	
H31	1.1867	0.4515	0.4575	0.055*	
C32	1.0393 (8)	0.4507 (5)	0.3997 (4)	0.046 (2)	
C33	0.9581 (8)	0.4048 (5)	0.3554 (4)	0.044 (2)	
H33	0.8959	0.4268	0.3374	0.053*	
C34	0.8736 (6)	0.2733 (4)	0.2891 (4)	0.041 (2)	
H34A	0.8043	0.2584	0.3088	0.049*	
H34B	0.8531	0.3051	0.2555	0.049*	
C35	0.9587 (8)	0.2094 (5)	0.2050 (4)	0.050 (2)	
H35	0.9031	0.2337	0.1786	0.060*	
C36	0.9599 (9)	0.1281 (5)	0.1695 (5)	0.073 (3)	
H36A	0.8844	0.0938	0.1677	0.088*	
H36B	1.0177	0.1018	0.1916	0.088*	
C37	0.9880 (11)	0.1373 (7)	0.1029 (5)	0.085 (4)	
H37A	0.9915	0.0845	0.0824	0.102*	
H37B	0.9259	0.1584	0.0795	0.102*	
C38	1.0978 (11)	0.1911 (7)	0.1021 (5)	0.090 (4)	
H38	1.1585	0.1653	0.1232	0.108*	
C39	1.1043 (9)	0.2720 (7)	0.1394 (5)	0.087 (3)	
H39A	1.0528	0.3029	0.1172	0.104*	
H39B	1.1832	0.3021	0.1433	0.104*	
C40	1.0688 (9)	0.2624 (6)	0.2064 (5)	0.083 (3)	
H40A	1.1290	0.2412	0.2313	0.100*	
H40B	1.0642	0.3153	0.2263	0.100*	
C41	0.9815 (6)	0.1804 (4)	0.6651 (3)	0.0342 (19)	
C42	1.0596 (7)	0.1269 (5)	0.6652 (4)	0.043 (2)	
C43	1.1287 (7)	0.1243 (5)	0.6175 (4)	0.054 (2)	
H43	1.1781	0.0864	0.6160	0.064*	
C44	1.1247 (7)	0.1775 (6)	0.5724 (4)	0.053 (2)	
H44	1.1722	0.1759	0.5411	0.063*	
C45	1.0516 (9)	0.2321 (5)	0.5737 (4)	0.059 (3)	
H45	1.0511	0.2690	0.5441	0.071*	
C46	0.9789 (8)	0.2331 (5)	0.6182 (4)	0.049 (2)	
H46	0.9268	0.2693	0.6175	0.059*	
C47	0.8971 (6)	0.1786 (4)	0.7138 (4)	0.038 (2)	
H47	0.8362	0.1309	0.7014	0.046*	
C48	1.0032 (7)	0.3208 (5)	0.7885 (4)	0.037 (2)	
C49	0.9029 (8)	0.3203 (5)	0.7471 (4)	0.042 (2)	
C50	0.8687 (8)	0.3950 (6)	0.7391 (4)	0.054 (2)	
C51	0.9346 (10)	0.4662 (5)	0.7697 (5)	0.072 (3)	
H51	0.9122	0.5158	0.7636	0.086*	
C52	1.0323 (10)	0.4621 (6)	0.8085 (5)	0.070 (3)	
C53	1.0690 (8)	0.3894 (6)	0.8191 (4)	0.054 (2)	
H53	1.1361	0.3879	0.8462	0.065*	
C54	1.0416 (7)	0.2411 (5)	0.7979 (4)	0.047 (2)	
H54A	1.1106	0.2392	0.7777	0.056*	
H54B	1.0643	0.2397	0.8422	0.056*	
C55	0.8793 (7)	0.1335 (5)	0.8192 (4)	0.049 (2)	
H55	0.8187	0.0928	0.7940	0.058*	
C56	0.9439 (8)	0.0884 (6)	0.8639 (4)	0.060 (3)	
H56A	1.0060	0.1262	0.8897	0.072*	
H56B	0.9794	0.0495	0.8408	0.072*	
C57	0.8643 (9)	0.0435 (6)	0.9062 (5)	0.076 (3)	
H57A	0.8087	0.0003	0.8814	0.091*	
H57B	0.9109	0.0195	0.9371	0.091*	
C58	0.8021 (8)	0.0999 (6)	0.9374 (4)	0.063 (3)	
H58	0.8590	0.1404	0.9650	0.075*	
C59	0.7345 (8)	0.1427 (6)	0.8917 (5)	0.069 (3)	
H59A	0.6948	0.1799	0.9139	0.083*	
H59B	0.6761	0.1035	0.8647	0.083*	
C60	0.8170 (8)	0.1891 (6)	0.8529 (4)	0.067 (3)	
H60A	0.7730	0.2165	0.8232	0.081*	
H60B	0.8730	0.2299	0.8799	0.081*	
C61	0.4694 (6)	0.1857 (5)	0.6699 (4)	0.036 (2)	
C62	0.5409 (7)	0.1278 (5)	0.6708 (4)	0.038 (2)	
C63	0.6068 (7)	0.1153 (5)	0.6243 (4)	0.051 (2)	
H63	0.6530	0.0754	0.6251	0.062*	
C64	0.6023 (9)	0.1646 (6)	0.5755 (5)	0.068 (3)	
H64	0.6441	0.1557	0.5425	0.082*	
C65	0.5389 (8)	0.2247 (6)	0.5752 (4)	0.059 (3)	
H65	0.5407	0.2589	0.5436	0.071*	
C66	0.4717 (7)	0.2349 (5)	0.6218 (4)	0.045 (2)	
H66	0.4268	0.2756	0.6211	0.054*	
C67	0.3914 (7)	0.1941 (5)	0.7208 (4)	0.040 (2)	
H67	0.3289	0.1466	0.7140	0.049*	
C68	0.5023 (7)	0.3429 (5)	0.7885 (4)	0.045 (2)	
C69	0.3967 (7)	0.3390 (5)	0.7479 (4)	0.039 (2)	
C70	0.3564 (8)	0.4105 (6)	0.7393 (4)	0.055 (3)	
C71	0.4159 (9)	0.4848 (6)	0.7707 (5)	0.070 (3)	
H71	0.3880	0.5324	0.7642	0.084*	
C72	0.5144 (10)	0.4863 (5)	0.8106 (5)	0.072 (3)	
C73	0.5602 (8)	0.4159 (6)	0.8211 (4)	0.058 (3)	
H73	0.6274	0.4179	0.8490	0.070*	
C74	0.5474 (7)	0.2652 (5)	0.7962 (4)	0.043 (2)	
H74A	0.6064	0.2613	0.7689	0.052*	
H74B	0.5846	0.2670	0.8387	0.052*	
C75	0.3780 (7)	0.1742 (5)	0.8313 (4)	0.043 (2)	
H75	0.3017	0.1864	0.8156	0.051*	
C76	0.3619 (8)	0.0868 (5)	0.8389 (4)	0.054 (2)	
H76A	0.4371	0.0728	0.8528	0.064*	
H76B	0.3323	0.0564	0.7987	0.064*	
C77	0.2793 (8)	0.0624 (5)	0.8848 (4)	0.062 (3)	
H77A	0.2766	0.0055	0.8900	0.074*	
H77B	0.2016	0.0697	0.8684	0.074*	
C78	0.3163 (7)	0.1119 (5)	0.9477 (4)	0.047 (2)	
H78	0.3919	0.1002	0.9653	0.057*	
C79	0.3307 (9)	0.1990 (5)	0.9392 (4)	0.070 (3)	
H79A	0.3580	0.2305	0.9791	0.084*	
H79B	0.2558	0.2121	0.9235	0.084*	
C80	0.4164 (9)	0.2215 (5)	0.8942 (4)	0.064 (3)	
H80A	0.4237	0.2789	0.8898	0.077*	
H80B	0.4924	0.2111	0.9109	0.077*	
O9	0.0335 (12)	0.3116 (7)	0.9790 (6)	0.072 (4)	0.50
H9	−0.0263	0.3256	0.9886	0.108*	0.50
O10	0.7990 (12)	0.3078 (8)	1.0151 (6)	0.081 (4)	0.50
H10	0.7985	0.2663	0.9924	0.121*	0.50
C81	0.1269 (17)	0.3789 (11)	0.9909 (10)	0.104 (8)	0.50
H81A	0.0960	0.4283	0.9913	0.155*	0.50
H81B	0.1763	0.3771	0.9588	0.155*	0.50
H81C	0.1715	0.3768	1.0306	0.155*	0.50
C82	0.7119 (18)	0.3499 (13)	0.9890 (11)	0.114 (9)	0.50
H82A	0.6939	0.3871	1.0208	0.170*	0.50
H82B	0.6428	0.3117	0.9723	0.170*	0.50
H82C	0.7404	0.3792	0.9562	0.170*	0.50
H1A	0.609 (7)	0.178 (5)	0.366 (3)	0.080*	
H3B	1.117 (6)	0.184 (4)	0.363 (4)	0.080*	
H5B	0.783 (5)	0.245 (5)	0.685 (3)	0.080*	
H7B	0.278 (5)	0.269 (5)	0.689 (3)	0.080*	
H7	0.528 (8)	0.100 (5)	0.751 (2)	0.080*	
H8	0.193 (7)	0.117 (5)	1.008 (4)	0.080*	

### Atomic displacement parameters (Å^2^)

**Table:**

	*U*^11^	*U*^22^	*U*^33^	*U*^12^	*U*^13^	*U*^23^
Br1	0.1302 (11)	0.0416 (6)	0.0861 (9)	0.0253 (6)	0.0123 (8)	−0.0075 (6)
Br2	0.0457 (6)	0.0728 (7)	0.0474 (6)	0.0083 (5)	−0.0034 (5)	0.0044 (5)
Br3	0.1229 (10)	0.0366 (6)	0.0691 (8)	0.0169 (6)	0.0102 (7)	−0.0075 (5)
Br4	0.0435 (6)	0.0741 (7)	0.0513 (6)	0.0068 (5)	−0.0033 (5)	0.0104 (5)
Br5	0.0702 (7)	0.0785 (8)	0.0846 (8)	0.0348 (6)	0.0184 (6)	0.0329 (6)
Br6	0.1881 (16)	0.0526 (8)	0.1468 (14)	−0.0179 (9)	−0.0318 (12)	−0.0100 (8)
Br7	0.0655 (7)	0.0694 (7)	0.0756 (8)	0.0291 (6)	0.0034 (6)	0.0201 (6)
Br8	0.1732 (15)	0.0526 (8)	0.1521 (14)	−0.0194 (8)	−0.0530 (12)	0.0089 (8)
O1	0.052 (4)	0.052 (4)	0.051 (4)	0.001 (3)	−0.006 (3)	0.000 (3)
O2	0.154 (8)	0.122 (8)	0.085 (6)	0.064 (6)	0.080 (6)	0.035 (5)
O3	0.058 (4)	0.061 (4)	0.045 (4)	0.000 (3)	−0.004 (3)	0.008 (3)
O4	0.113 (7)	0.093 (6)	0.057 (5)	0.014 (5)	0.050 (4)	0.002 (4)
O5	0.068 (5)	0.067 (4)	0.056 (5)	0.030 (4)	0.017 (3)	0.016 (4)
O6	0.090 (5)	0.075 (5)	0.076 (5)	0.019 (4)	0.047 (4)	0.034 (4)
O7	0.067 (4)	0.068 (4)	0.056 (5)	0.032 (4)	0.020 (4)	0.010 (4)
O8	0.076 (5)	0.085 (5)	0.075 (5)	0.021 (4)	0.059 (4)	0.027 (4)
N1	0.033 (4)	0.035 (4)	0.052 (5)	0.004 (3)	0.003 (4)	0.004 (3)
N2	0.043 (4)	0.035 (4)	0.030 (4)	0.003 (3)	0.005 (3)	−0.003 (3)
N3	0.044 (5)	0.034 (4)	0.044 (5)	0.008 (3)	0.005 (4)	0.002 (3)
N4	0.045 (4)	0.033 (4)	0.035 (4)	0.015 (3)	0.003 (3)	0.005 (3)
N5	0.039 (4)	0.051 (5)	0.040 (5)	0.011 (4)	0.002 (3)	0.007 (4)
N6	0.030 (4)	0.037 (4)	0.035 (4)	−0.001 (3)	0.006 (3)	0.011 (3)
N7	0.052 (5)	0.044 (4)	0.045 (5)	0.014 (4)	0.009 (4)	0.006 (4)
N8	0.036 (4)	0.049 (4)	0.036 (4)	0.009 (3)	0.012 (3)	0.009 (3)
C1	0.043 (5)	0.030 (4)	0.031 (5)	0.012 (4)	0.008 (4)	0.001 (4)
C2	0.047 (6)	0.029 (5)	0.064 (7)	0.017 (4)	0.019 (5)	0.009 (4)
C3	0.052 (6)	0.019 (4)	0.090 (8)	0.005 (4)	0.021 (6)	0.019 (5)
C4	0.063 (7)	0.043 (6)	0.067 (8)	0.011 (5)	0.034 (6)	0.028 (5)
C5	0.069 (7)	0.052 (6)	0.046 (6)	0.006 (5)	0.017 (6)	0.013 (5)
C6	0.043 (5)	0.046 (5)	0.053 (6)	0.003 (4)	0.011 (5)	0.007 (5)
C7	0.037 (5)	0.038 (5)	0.038 (5)	0.013 (4)	0.010 (4)	0.003 (4)
C8	0.047 (5)	0.034 (5)	0.031 (5)	0.016 (4)	0.010 (4)	0.009 (4)
C9	0.032 (5)	0.029 (5)	0.036 (5)	−0.001 (4)	0.006 (4)	0.006 (4)
C10	0.038 (5)	0.033 (5)	0.045 (6)	0.005 (4)	0.016 (4)	−0.002 (4)
C11	0.063 (7)	0.047 (6)	0.038 (6)	−0.012 (5)	0.006 (5)	−0.001 (4)
C12	0.074 (7)	0.028 (5)	0.047 (6)	0.013 (5)	0.009 (5)	0.001 (4)
C13	0.054 (6)	0.048 (6)	0.048 (6)	0.023 (5)	0.020 (5)	0.015 (5)
C14	0.045 (5)	0.052 (6)	0.042 (6)	0.006 (4)	−0.004 (4)	0.003 (4)
C15	0.060 (6)	0.050 (6)	0.031 (5)	0.004 (5)	0.012 (5)	0.009 (4)
C16	0.069 (7)	0.055 (6)	0.037 (6)	−0.006 (5)	0.011 (5)	−0.005 (4)
C17	0.094 (8)	0.051 (6)	0.064 (7)	0.000 (6)	0.027 (6)	0.004 (5)
C18	0.074 (8)	0.074 (8)	0.057 (7)	0.003 (6)	0.011 (6)	−0.008 (6)
C19	0.075 (7)	0.052 (6)	0.055 (7)	0.001 (5)	0.019 (5)	−0.005 (5)
C20	0.093 (8)	0.044 (6)	0.052 (7)	−0.011 (5)	0.034 (6)	−0.006 (5)
C21	0.045 (5)	0.023 (4)	0.046 (6)	0.015 (4)	0.000 (4)	0.006 (4)
C22	0.049 (6)	0.030 (5)	0.050 (6)	0.007 (4)	−0.001 (5)	0.005 (4)
C23	0.044 (6)	0.040 (5)	0.074 (8)	−0.001 (4)	0.014 (6)	0.022 (5)
C24	0.060 (7)	0.050 (6)	0.070 (8)	0.015 (5)	0.029 (6)	0.027 (5)
C25	0.074 (8)	0.049 (6)	0.048 (6)	0.011 (5)	0.025 (6)	0.017 (5)
C26	0.054 (6)	0.045 (5)	0.042 (6)	0.015 (5)	−0.010 (5)	0.000 (4)
C27	0.038 (5)	0.023 (4)	0.043 (5)	0.000 (4)	0.000 (4)	−0.005 (4)
C28	0.038 (5)	0.032 (5)	0.039 (5)	0.000 (4)	0.004 (4)	−0.004 (4)
C29	0.024 (4)	0.033 (5)	0.039 (5)	0.002 (4)	0.014 (4)	−0.003 (4)
C30	0.037 (5)	0.049 (6)	0.039 (5)	0.008 (4)	0.011 (4)	0.012 (4)
C31	0.058 (6)	0.036 (5)	0.036 (5)	−0.006 (5)	−0.002 (5)	0.004 (4)
C32	0.072 (7)	0.030 (5)	0.038 (5)	0.011 (5)	0.013 (5)	−0.001 (4)
C33	0.065 (6)	0.040 (5)	0.029 (5)	0.011 (5)	0.016 (5)	0.003 (4)
C34	0.037 (5)	0.039 (5)	0.051 (6)	0.008 (4)	0.012 (4)	0.018 (4)
C35	0.072 (7)	0.037 (5)	0.042 (6)	0.009 (5)	0.014 (5)	0.006 (4)
C36	0.094 (8)	0.057 (7)	0.066 (8)	0.007 (6)	0.020 (6)	−0.008 (5)
C37	0.123 (11)	0.091 (9)	0.052 (7)	0.051 (8)	0.019 (7)	0.004 (6)
C38	0.099 (10)	0.087 (9)	0.094 (10)	0.015 (7)	0.068 (8)	−0.005 (7)
C39	0.084 (8)	0.100 (9)	0.076 (8)	−0.005 (7)	0.029 (7)	0.018 (7)
C40	0.084 (8)	0.093 (9)	0.064 (8)	−0.017 (7)	0.026 (6)	0.008 (6)
C41	0.036 (5)	0.034 (5)	0.030 (5)	−0.002 (4)	0.008 (4)	−0.010 (4)
C42	0.038 (5)	0.042 (5)	0.050 (6)	0.013 (4)	0.013 (5)	−0.007 (4)
C43	0.050 (6)	0.055 (6)	0.058 (7)	0.012 (5)	0.019 (5)	−0.003 (5)
C44	0.050 (6)	0.075 (7)	0.033 (6)	0.007 (5)	0.021 (5)	−0.003 (5)
C45	0.101 (8)	0.048 (6)	0.040 (6)	0.022 (6)	0.030 (6)	0.020 (5)
C46	0.074 (7)	0.040 (5)	0.032 (5)	0.003 (5)	0.018 (5)	−0.003 (4)
C47	0.033 (5)	0.029 (4)	0.057 (6)	0.010 (4)	0.011 (4)	0.013 (4)
C48	0.052 (6)	0.033 (5)	0.033 (5)	0.015 (4)	0.014 (4)	0.006 (4)
C49	0.051 (6)	0.037 (5)	0.040 (6)	0.000 (5)	0.018 (5)	0.005 (4)
C50	0.063 (7)	0.052 (6)	0.048 (6)	0.007 (5)	0.008 (5)	0.012 (5)
C51	0.117 (10)	0.030 (6)	0.076 (8)	0.018 (6)	0.033 (7)	0.015 (5)
C52	0.088 (8)	0.060 (7)	0.046 (7)	−0.013 (6)	−0.006 (6)	−0.005 (5)
C53	0.061 (6)	0.049 (6)	0.051 (6)	0.002 (5)	0.013 (5)	0.009 (5)
C54	0.044 (5)	0.061 (6)	0.035 (5)	0.006 (5)	0.010 (4)	0.004 (4)
C55	0.055 (6)	0.061 (6)	0.037 (5)	0.019 (5)	0.013 (5)	0.020 (5)
C56	0.062 (6)	0.076 (7)	0.054 (6)	0.025 (5)	0.029 (5)	0.028 (5)
C57	0.096 (8)	0.067 (7)	0.079 (8)	0.027 (6)	0.035 (7)	0.025 (6)
C58	0.074 (7)	0.073 (7)	0.053 (7)	0.024 (6)	0.025 (6)	0.031 (5)
C59	0.056 (6)	0.100 (8)	0.076 (7)	0.041 (6)	0.044 (6)	0.047 (6)
C60	0.073 (7)	0.084 (7)	0.064 (7)	0.045 (6)	0.025 (6)	0.027 (6)
C61	0.033 (5)	0.037 (5)	0.041 (5)	0.006 (4)	0.013 (4)	0.007 (4)
C62	0.043 (5)	0.038 (5)	0.034 (5)	0.004 (4)	0.007 (4)	0.004 (4)
C63	0.052 (6)	0.055 (6)	0.046 (6)	0.013 (5)	0.010 (5)	−0.005 (5)
C64	0.071 (7)	0.079 (8)	0.048 (7)	−0.009 (6)	0.028 (6)	−0.021 (6)
C65	0.085 (8)	0.053 (6)	0.046 (6)	0.018 (6)	0.022 (6)	0.013 (5)
C66	0.057 (6)	0.048 (5)	0.035 (5)	0.019 (5)	0.010 (5)	0.009 (4)
C67	0.039 (5)	0.041 (5)	0.042 (6)	0.010 (4)	0.007 (4)	−0.002 (4)
C68	0.032 (5)	0.066 (6)	0.038 (5)	0.005 (5)	0.009 (4)	0.017 (5)
C69	0.037 (5)	0.049 (6)	0.038 (5)	0.018 (4)	0.016 (4)	0.011 (4)
C70	0.049 (6)	0.071 (7)	0.056 (7)	0.018 (5)	0.023 (5)	0.030 (5)
C71	0.077 (8)	0.045 (6)	0.089 (9)	0.018 (6)	−0.004 (7)	0.015 (6)
C72	0.086 (8)	0.038 (6)	0.083 (8)	−0.011 (6)	−0.006 (7)	0.014 (5)
C73	0.053 (6)	0.064 (7)	0.048 (6)	−0.016 (5)	−0.007 (5)	0.020 (5)
C74	0.033 (5)	0.069 (6)	0.035 (5)	0.016 (5)	0.020 (4)	0.009 (4)
C75	0.034 (5)	0.066 (6)	0.036 (5)	0.020 (4)	0.019 (4)	0.015 (4)
C76	0.069 (7)	0.048 (6)	0.040 (6)	−0.009 (5)	0.016 (5)	0.008 (4)
C77	0.069 (7)	0.056 (6)	0.064 (7)	−0.002 (5)	0.033 (6)	0.015 (5)
C78	0.049 (6)	0.056 (6)	0.045 (6)	0.014 (5)	0.023 (5)	0.014 (5)
C79	0.124 (9)	0.043 (6)	0.056 (7)	0.019 (6)	0.053 (6)	0.012 (5)
C80	0.110 (8)	0.047 (6)	0.043 (6)	0.014 (5)	0.041 (6)	0.004 (5)
O9	0.096 (8)	0.065 (7)	0.053 (7)	0.024 (6)	−0.009 (6)	0.006 (6)
O10	0.094 (8)	0.065 (7)	0.075 (8)	−0.006 (6)	0.015 (7)	−0.006 (6)
C81	0.127 (11)	0.108 (11)	0.080 (10)	0.022 (9)	0.009 (8)	0.043 (8)
C82	0.124 (12)	0.111 (12)	0.100 (12)	0.018 (9)	0.005 (9)	−0.003 (9)

### Geometric parameters (Å, °)

**Table:**

Br1—C12	1.907 (8)	C33—H33	0.9300
Br2—C10	1.902 (8)	C34—H34A	0.9700
Br3—C32	1.903 (7)	C34—H34B	0.9700
Br4—C30	1.920 (8)	C35—C40	1.440 (11)
Br5—C50	1.902 (9)	C35—C36	1.518 (11)
Br6—C52	1.911 (9)	C35—H35	0.9800
Br7—C70	1.872 (9)	C36—C37	1.537 (12)
Br8—C72	1.892 (9)	C36—H36A	0.9700
O1—C22	1.376 (9)	C36—H36B	0.9700
O1—H1	0.8200	C37—C38	1.450 (14)
O2—C38	1.429 (11)	C37—H37A	0.9700
O2—H2	0.8200	C37—H37B	0.9700
O3—C2	1.353 (9)	C38—C39	1.513 (13)
O3—H3	0.8200	C38—H38	0.9800
O4—C18	1.425 (10)	C39—C40	1.575 (12)
O4—H4	0.8200	C39—H39A	0.9700
O5—C42	1.359 (9)	C39—H39B	0.9700
O5—H5	0.8200	C40—H40A	0.9700
O6—C58	1.458 (10)	C40—H40B	0.9700
O6—H6	0.8200	C41—C42	1.387 (10)
O7—C62	1.381 (9)	C41—C46	1.405 (10)
O7—H7	0.85 (6)	C41—C47	1.531 (10)
O8—C78	1.419 (9)	C42—C43	1.392 (11)
O8—H8	0.84 (9)	C43—C44	1.382 (11)
N1—C9	1.373 (9)	C43—H43	0.9300
N1—C7	1.467 (9)	C44—C45	1.356 (11)
N1—H1A	0.91 (8)	C44—H44	0.9300
N2—C14	1.468 (9)	C45—C46	1.365 (11)
N2—C15	1.475 (9)	C45—H45	0.9300
N2—C7	1.477 (9)	C46—H46	0.9300
N3—C29	1.394 (9)	C47—H47	0.9800
N3—C27	1.462 (9)	C48—C53	1.352 (11)
N3—H3B	0.91 (8)	C48—C49	1.391 (11)
N4—C27	1.446 (9)	C48—C54	1.506 (10)
N4—C35	1.459 (9)	C49—C50	1.400 (11)
N4—C34	1.465 (9)	C50—C51	1.384 (12)
N5—C49	1.367 (10)	C51—C52	1.355 (13)
N5—C47	1.470 (9)	C51—H51	0.9300
N5—H5B	0.90 (6)	C52—C53	1.390 (12)
N6—C47	1.462 (9)	C53—H53	0.9300
N6—C54	1.464 (9)	C54—H54A	0.9700
N6—C55	1.485 (9)	C54—H54B	0.9700
N7—C69	1.376 (10)	C55—C60	1.480 (11)
N7—C67	1.460 (9)	C55—C56	1.493 (10)
N7—H7B	0.90 (6)	C55—H55	0.9800
N8—C74	1.463 (9)	C56—C57	1.541 (11)
N8—C67	1.469 (9)	C56—H56A	0.9700
N8—C75	1.513 (9)	C56—H56B	0.9700
C1—C6	1.386 (10)	C57—C58	1.460 (11)
C1—C2	1.411 (11)	C57—H57A	0.9700
C1—C7	1.529 (10)	C57—H57B	0.9700
C2—C3	1.403 (11)	C58—C59	1.498 (11)
C3—C4	1.378 (12)	C58—H58	0.9800
C3—H3A	0.9300	C59—C60	1.521 (11)
C4—C5	1.367 (12)	C59—H59A	0.9700
C4—H4A	0.9300	C59—H59B	0.9700
C5—C6	1.361 (11)	C60—H60A	0.9700
C5—H5A	0.9300	C60—H60B	0.9700
C6—H6A	0.9300	C61—C66	1.385 (10)
C7—H7A	0.9800	C61—C62	1.385 (10)
C8—C13	1.379 (10)	C61—C67	1.529 (10)
C8—C9	1.411 (10)	C62—C63	1.365 (11)
C8—C14	1.498 (10)	C63—C64	1.400 (12)
C9—C10	1.395 (10)	C63—H63	0.9300
C10—C11	1.375 (10)	C64—C65	1.351 (12)
C11—C12	1.343 (11)	C64—H64	0.9300
C11—H11	0.9300	C65—C66	1.372 (11)
C12—C13	1.387 (11)	C65—H65	0.9300
C13—H13	0.9300	C66—H66	0.9300
C14—H14A	0.9700	C67—H67	0.9800
C14—H14B	0.9700	C68—C73	1.394 (11)
C15—C20	1.455 (11)	C68—C69	1.421 (10)
C15—C16	1.515 (10)	C68—C74	1.502 (11)
C15—H15	0.9800	C69—C70	1.381 (11)
C16—C17	1.538 (11)	C70—C71	1.406 (12)
C16—H16A	0.9700	C71—C72	1.353 (12)
C16—H16B	0.9700	C71—H71	0.9300
C17—C18	1.453 (12)	C72—C73	1.403 (12)
C17—H17A	0.9700	C73—H73	0.9300
C17—H17B	0.9700	C74—H74A	0.9700
C18—C19	1.505 (11)	C74—H74B	0.9700
C18—H18	0.9800	C75—C76	1.477 (10)
C19—C20	1.508 (11)	C75—C80	1.503 (11)
C19—H19A	0.9700	C75—H75	0.9800
C19—H19B	0.9700	C76—C77	1.506 (10)
C20—H20A	0.9700	C76—H76A	0.9700
C20—H20B	0.9700	C76—H76B	0.9700
C21—C22	1.398 (11)	C77—C78	1.518 (11)
C21—C26	1.411 (10)	C77—H77A	0.9700
C21—C27	1.530 (10)	C77—H77B	0.9700
C22—C23	1.364 (11)	C78—C79	1.476 (10)
C23—C24	1.375 (12)	C78—H78	0.9800
C23—H23	0.9300	C79—C80	1.510 (11)
C24—C25	1.351 (12)	C79—H79A	0.9700
C24—H24	0.9300	C79—H79B	0.9700
C25—C26	1.361 (11)	C80—H80A	0.9700
C25—H25	0.9300	C80—H80B	0.9700
C26—H26	0.9300	O9—C81	1.425 (10)
C27—H27	0.9800	O9—H9	0.8200
C28—C33	1.371 (10)	O10—C82	1.427 (9)
C28—C29	1.392 (10)	O10—H10	0.8200
C28—C34	1.526 (10)	C81—H81A	0.9600
C29—C30	1.374 (10)	C81—H81B	0.9600
C30—C31	1.380 (10)	C81—H81C	0.9600
C31—C32	1.367 (11)	C82—H82A	0.9600
C31—H31	0.9300	C82—H82B	0.9600
C32—C33	1.360 (11)	C82—H82C	0.9600
			
C22—O1—H1	109.5	C38—C39—H39B	109.2
C38—O2—H2	109.5	C40—C39—H39B	109.2
C2—O3—H3	109.5	H39A—C39—H39B	107.9
C18—O4—H4	109.5	C35—C40—C39	112.6 (9)
C42—O5—H5	109.5	C35—C40—H40A	109.1
C58—O6—H6	109.5	C39—C40—H40A	109.1
C62—O7—H7	110 (7)	C35—C40—H40B	109.1
C78—O8—H8	131 (7)	C39—C40—H40B	109.1
C9—N1—C7	117.4 (6)	H40A—C40—H40B	107.8
C9—N1—H1A	120 (6)	C42—C41—C46	118.5 (8)
C7—N1—H1A	109 (6)	C42—C41—C47	119.9 (7)
C14—N2—C15	115.1 (6)	C46—C41—C47	121.6 (7)
C14—N2—C7	109.9 (6)	O5—C42—C41	122.8 (8)
C15—N2—C7	117.2 (6)	O5—C42—C43	117.9 (7)
C29—N3—C27	117.7 (6)	C41—C42—C43	119.3 (8)
C29—N3—H3B	126 (6)	C44—C43—C42	120.6 (8)
C27—N3—H3B	108 (6)	C44—C43—H43	119.7
C27—N4—C35	117.6 (6)	C42—C43—H43	119.7
C27—N4—C34	110.5 (6)	C45—C44—C43	120.1 (8)
C35—N4—C34	113.2 (6)	C45—C44—H44	120.0
C49—N5—C47	117.2 (7)	C43—C44—H44	120.0
C49—N5—H5B	125 (6)	C44—C45—C46	120.4 (8)
C47—N5—H5B	109 (6)	C44—C45—H45	119.8
C47—N6—C54	109.0 (6)	C46—C45—H45	119.8
C47—N6—C55	115.4 (6)	C45—C46—C41	121.0 (8)
C54—N6—C55	117.2 (6)	C45—C46—H46	119.5
C69—N7—C67	119.5 (7)	C41—C46—H46	119.5
C69—N7—H7B	115 (6)	N6—C47—N5	110.7 (6)
C67—N7—H7B	121 (6)	N6—C47—C41	110.5 (6)
C74—N8—C67	109.2 (6)	N5—C47—C41	114.2 (6)
C74—N8—C75	116.9 (6)	N6—C47—H47	107.0
C67—N8—C75	111.9 (6)	N5—C47—H47	107.0
C6—C1—C2	117.3 (8)	C41—C47—H47	107.0
C6—C1—C7	121.4 (7)	C53—C48—C49	123.2 (8)
C2—C1—C7	121.3 (7)	C53—C48—C54	118.8 (8)
O3—C2—C3	119.6 (8)	C49—C48—C54	118.0 (7)
O3—C2—C1	120.9 (8)	N5—C49—C48	121.1 (8)
C3—C2—C1	119.5 (9)	N5—C49—C50	121.8 (8)
C4—C3—C2	119.3 (9)	C48—C49—C50	117.0 (8)
C4—C3—H3A	120.3	C51—C50—C49	121.0 (9)
C2—C3—H3A	120.3	C51—C50—Br5	119.0 (8)
C5—C4—C3	122.1 (9)	C49—C50—Br5	120.0 (7)
C5—C4—H4A	119.0	C52—C51—C50	118.7 (9)
C3—C4—H4A	119.0	C52—C51—H51	120.7
C6—C5—C4	118.1 (9)	C50—C51—H51	120.7
C6—C5—H5A	121.0	C51—C52—C53	122.7 (9)
C4—C5—H5A	121.0	C51—C52—Br6	118.0 (8)
C5—C6—C1	123.7 (8)	C53—C52—Br6	119.3 (8)
C5—C6—H6A	118.2	C48—C53—C52	117.4 (9)
C1—C6—H6A	118.2	C48—C53—H53	121.3
N1—C7—N2	111.0 (6)	C52—C53—H53	121.3
N1—C7—C1	113.9 (6)	N6—C54—C48	116.7 (7)
N2—C7—C1	110.4 (6)	N6—C54—H54A	108.1
N1—C7—H7A	107.1	C48—C54—H54A	108.1
N2—C7—H7A	107.1	N6—C54—H54B	108.1
C1—C7—H7A	107.1	C48—C54—H54B	108.1
C13—C8—C9	118.5 (8)	H54A—C54—H54B	107.3
C13—C8—C14	121.4 (7)	C60—C55—N6	117.7 (7)
C9—C8—C14	119.7 (7)	C60—C55—C56	110.4 (7)
N1—C9—C10	121.9 (7)	N6—C55—C56	110.4 (7)
N1—C9—C8	119.4 (7)	C60—C55—H55	105.9
C10—C9—C8	118.5 (7)	N6—C55—H55	105.9
C11—C10—C9	121.3 (8)	C56—C55—H55	105.9
C11—C10—Br2	119.9 (6)	C55—C56—C57	112.5 (7)
C9—C10—Br2	118.8 (6)	C55—C56—H56A	109.1
C12—C11—C10	120.0 (8)	C57—C56—H56A	109.1
C12—C11—H11	120.0	C55—C56—H56B	109.1
C10—C11—H11	120.0	C57—C56—H56B	109.1
C11—C12—C13	120.5 (8)	H56A—C56—H56B	107.8
C11—C12—Br1	119.7 (7)	C58—C57—C56	109.9 (8)
C13—C12—Br1	119.7 (7)	C58—C57—H57A	109.7
C8—C13—C12	121.2 (8)	C56—C57—H57A	109.7
C8—C13—H13	119.4	C58—C57—H57B	109.7
C12—C13—H13	119.4	C56—C57—H57B	109.7
N2—C14—C8	115.0 (6)	H57A—C57—H57B	108.2
N2—C14—H14A	108.5	O6—C58—C57	109.3 (8)
C8—C14—H14A	108.5	O6—C58—C59	109.6 (7)
N2—C14—H14B	108.5	C57—C58—C59	111.9 (8)
C8—C14—H14B	108.5	O6—C58—H58	108.6
H14A—C14—H14B	107.5	C57—C58—H58	108.6
C20—C15—N2	119.7 (7)	C59—C58—H58	108.6
C20—C15—C16	112.3 (7)	C58—C59—C60	109.7 (7)
N2—C15—C16	111.3 (7)	C58—C59—H59A	109.7
C20—C15—H15	103.8	C60—C59—H59A	109.7
N2—C15—H15	103.8	C58—C59—H59B	109.7
C16—C15—H15	103.8	C60—C59—H59B	109.7
C15—C16—C17	110.1 (7)	H59A—C59—H59B	108.2
C15—C16—H16A	109.6	C55—C60—C59	110.6 (8)
C17—C16—H16A	109.6	C55—C60—H60A	109.5
C15—C16—H16B	109.6	C59—C60—H60A	109.5
C17—C16—H16B	109.6	C55—C60—H60B	109.5
H16A—C16—H16B	108.1	C59—C60—H60B	109.5
C18—C17—C16	114.1 (8)	H60A—C60—H60B	108.1
C18—C17—H17A	108.7	C66—C61—C62	117.7 (7)
C16—C17—H17A	108.7	C66—C61—C67	121.9 (7)
C18—C17—H17B	108.7	C62—C61—C67	120.4 (7)
C16—C17—H17B	108.7	C63—C62—O7	115.8 (8)
H17A—C17—H17B	107.6	C63—C62—C61	122.1 (8)
O4—C18—C17	110.9 (8)	O7—C62—C61	122.0 (7)
O4—C18—C19	112.4 (8)	C62—C63—C64	117.8 (9)
C17—C18—C19	112.2 (8)	C62—C63—H63	121.1
O4—C18—H18	107.0	C64—C63—H63	121.1
C17—C18—H18	107.0	C65—C64—C63	121.5 (9)
C19—C18—H18	107.0	C65—C64—H64	119.3
C18—C19—C20	112.1 (8)	C63—C64—H64	119.3
C18—C19—H19A	109.2	C64—C65—C66	119.4 (9)
C20—C19—H19A	109.2	C64—C65—H65	120.3
C18—C19—H19B	109.2	C66—C65—H65	120.3
C20—C19—H19B	109.2	C65—C66—C61	121.3 (8)
H19A—C19—H19B	107.9	C65—C66—H66	119.4
C15—C20—C19	113.3 (8)	C61—C66—H66	119.4
C15—C20—H20A	108.9	N7—C67—N8	110.6 (6)
C19—C20—H20A	108.9	N7—C67—C61	113.9 (7)
C15—C20—H20B	108.9	N8—C67—C61	110.1 (6)
C19—C20—H20B	108.9	N7—C67—H67	107.3
H20A—C20—H20B	107.7	N8—C67—H67	107.3
C22—C21—C26	115.8 (8)	C61—C67—H67	107.3
C22—C21—C27	120.8 (8)	C73—C68—C69	120.9 (8)
C26—C21—C27	123.3 (8)	C73—C68—C74	121.4 (8)
C23—C22—O1	118.7 (8)	C69—C68—C74	117.7 (8)
C23—C22—C21	121.3 (9)	N7—C69—C70	121.7 (8)
O1—C22—C21	120.0 (8)	N7—C69—C68	120.4 (7)
C22—C23—C24	120.6 (9)	C70—C69—C68	118.0 (8)
C22—C23—H23	119.7	C69—C70—C71	121.4 (9)
C24—C23—H23	119.7	C69—C70—Br7	121.0 (8)
C25—C24—C23	120.1 (9)	C71—C70—Br7	117.6 (7)
C25—C24—H24	119.9	C72—C71—C70	119.4 (9)
C23—C24—H24	119.9	C72—C71—H71	120.3
C24—C25—C26	120.0 (9)	C70—C71—H71	120.3
C24—C25—H25	120.0	C71—C72—C73	122.0 (9)
C26—C25—H25	120.0	C71—C72—Br8	121.5 (8)
C25—C26—C21	122.2 (9)	C73—C72—Br8	116.6 (8)
C25—C26—H26	118.9	C68—C73—C72	118.3 (8)
C21—C26—H26	118.9	C68—C73—H73	120.9
N4—C27—N3	111.8 (6)	C72—C73—H73	120.9
N4—C27—C21	110.5 (6)	N8—C74—C68	114.0 (6)
N3—C27—C21	113.3 (6)	N8—C74—H74A	108.7
N4—C27—H27	107.0	C68—C74—H74A	108.7
N3—C27—H27	107.0	N8—C74—H74B	108.7
C21—C27—H27	107.0	C68—C74—H74B	108.7
C33—C28—C29	120.0 (7)	H74A—C74—H74B	107.6
C33—C28—C34	121.2 (7)	C76—C75—C80	109.0 (7)
C29—C28—C34	118.8 (7)	C76—C75—N8	109.5 (6)
C30—C29—C28	117.7 (7)	C80—C75—N8	115.8 (7)
C30—C29—N3	122.4 (7)	C76—C75—H75	107.4
C28—C29—N3	119.9 (7)	C80—C75—H75	107.4
C29—C30—C31	122.6 (7)	N8—C75—H75	107.4
C29—C30—Br4	119.1 (6)	C75—C76—C77	113.1 (7)
C31—C30—Br4	118.2 (6)	C75—C76—H76A	109.0
C32—C31—C30	117.9 (8)	C77—C76—H76A	109.0
C32—C31—H31	121.0	C75—C76—H76B	109.0
C30—C31—H31	121.0	C77—C76—H76B	109.0
C33—C32—C31	121.1 (8)	H76A—C76—H76B	107.8
C33—C32—Br3	119.8 (7)	C76—C77—C78	111.8 (7)
C31—C32—Br3	119.1 (6)	C76—C77—H77A	109.3
C32—C33—C28	120.8 (8)	C78—C77—H77A	109.3
C32—C33—H33	119.6	C76—C77—H77B	109.3
C28—C33—H33	119.6	C78—C77—H77B	109.3
N4—C34—C28	115.3 (6)	H77A—C77—H77B	107.9
N4—C34—H34A	108.5	O8—C78—C79	112.2 (7)
C28—C34—H34A	108.5	O8—C78—C77	110.5 (7)
N4—C34—H34B	108.5	C79—C78—C77	109.3 (7)
C28—C34—H34B	108.5	O8—C78—H78	108.2
H34A—C34—H34B	107.5	C79—C78—H78	108.2
C40—C35—N4	120.3 (8)	C77—C78—H78	108.2
C40—C35—C36	110.4 (8)	C78—C79—C80	111.6 (7)
N4—C35—C36	111.1 (7)	C78—C79—H79A	109.3
C40—C35—H35	104.5	C80—C79—H79A	109.3
N4—C35—H35	104.5	C78—C79—H79B	109.3
C36—C35—H35	104.5	C80—C79—H79B	109.3
C35—C36—C37	111.7 (8)	H79A—C79—H79B	108.0
C35—C36—H36A	109.3	C75—C80—C79	111.4 (8)
C37—C36—H36A	109.3	C75—C80—H80A	109.3
C35—C36—H36B	109.3	C79—C80—H80A	109.3
C37—C36—H36B	109.3	C75—C80—H80B	109.3
H36A—C36—H36B	107.9	C79—C80—H80B	109.3
C38—C37—C36	112.1 (9)	H80A—C80—H80B	108.0
C38—C37—H37A	109.2	C81—O9—H9	109.5
C36—C37—H37A	109.2	C82—O10—H10	109.5
C38—C37—H37B	109.2	O9—C81—H81A	109.5
C36—C37—H37B	109.2	O9—C81—H81B	109.5
H37A—C37—H37B	107.9	H81A—C81—H81B	109.5
O2—C38—C37	116.8 (11)	O9—C81—H81C	109.5
O2—C38—C39	108.2 (10)	H81A—C81—H81C	109.5
C37—C38—C39	112.4 (9)	H81B—C81—H81C	109.5
O2—C38—H38	106.3	O10—C82—H82A	109.5
C37—C38—H38	106.3	O10—C82—H82B	109.5
C39—C38—H38	106.3	H82A—C82—H82B	109.5
C38—C39—C40	112.2 (9)	O10—C82—H82C	109.5
C38—C39—H39A	109.2	H82A—C82—H82C	109.5
C40—C39—H39A	109.2	H82B—C82—H82C	109.5

### Hydrogen-bond geometry (Å, °)

**Table:**

*D*—H···*A*	*D*—H	H···*A*	*D*···*A*	*D*—H···*A*
O1—H1···N4	0.82	1.93	2.643 (8)	145
N1—H1A···Br2	0.91 (8)	2.69 (7)	3.081 (7)	107 (6)
O2—H2···O9^i^	0.82	1.85	2.660 (15)	167
O3—H3···N2	0.82	1.93	2.661 (9)	148
N3—H3B···Br4	0.91 (8)	2.77 (8)	3.097 (7)	103 (5)
O4—H4···O6^ii^	0.82	2.13	2.721 (10)	129
O5—H5···N6	0.82	1.95	2.650 (9)	143
N5—H5B···Br5	0.90 (6)	2.77 (8)	3.110 (7)	104 (6)
O6—H6···O8^iii^	0.82	1.95	2.735 (9)	160
O7—H7···N8	0.85 (6)	1.99 (8)	2.717 (9)	142 (6)
N7—H7B···Br7	0.90 (6)	2.61 (8)	3.100 (7)	115 (6)
O8—H8···O2^iv^	0.84 (9)	1.90 (8)	2.713 (11)	162 (8)
O9—H9···O10^v^	0.82	2.18	2.94 (2)	156

Symmetry codes: (i) *x*+1, *y*, *z*−1; (ii) *x*, *y*, *z*−1; (iii) −*x*+1, −*y*, −*z*+2; (iv) *x*−1, *y*, *z*+1; (v) *x*−1, *y*, *z*.
